# Facteurs associés à l’infection de l’hépatite B chez les femmes enceintes dans les formations sanitaires du district de santé de Mokolo/Région de l’Extrême-Nord Cameroun

**DOI:** 10.11604/pamj.2022.41.61.32627

**Published:** 2022-01-21

**Authors:** Abdoul Rahamane Njigou Mawouma, Amadou Hapsatou Djoulatou, Eliane Ornella Komnang, Etienne Omolomo Kimessoukie

**Affiliations:** 1 École des Sciences de la Santé de l´Université Catholique d´Afrique Centrale, Yaoundé, Cameroun,; 2 Faculté de Médecine et des Sciences Biomédicales de l´Université de Yaoundé I, Yaoundé, Cameroun

**Keywords:** Hépatite B, femmes enceintes, facteurs associés, Cameroun, Hepatitis B, pregnant women, associated factors, Cameroon

## Abstract

**Introduction:**

la transmission périnatale de l´hépatite B est responsable de plus d´un tiers des hépatites virales dans les régions à forte endémicité. Cette étude vise à analyser les facteurs associés à l´infection de l´hépatite B chez les femmes enceintes dans le district de santé de Mokolo/Extrême-Nord Cameroun.

**Méthodes:**

une étude transversale a été mobilisée d´avril 2020 à janvier 2021. Un échantillon de 794 femmes enceintes consentantes a été constitué par quota et par convenance dans sept formations sanitaires du district de santé de Mokolo. Les données sociodémographiques et les antécédents ont été collectés. La recherche des marqueurs sériques de l´hépatite B, et les co-infections (Virus de l´Immunodéficience Humaine, syphilis et hépatite C) a été réalisée. Le degré d´association avec le portage de l´AgHbs a été recherché pour un seuil de signification fixé à 5%.

**Résultats:**

après l´analyse de régression logistique multivariée, le niveau d´étude (OR = 1,58 IC à 95% = 1,08-2,30 p = 0,019), la profession du partenaire (OR = 4,07 IC à 95% = 1,33-12,53 p = 0,014), le premier trimestre de la grossesse (OR = 2,38 IC à 95% = 1,12-5,04 p = 0,024) et la sérologie syphilis (OR = 3,27 IC à 95% = 1,29-8,28 p = 0,012) se sont révélés être des facteurs de risque majeurs. La séroprévalence de l´AgHbs est de 18,4% (IC à 95% = 15,9-21,3) avec une positivité à l´AgHbe de 13,7% (IC à 95% = 7,5-19,9) dans le district de santé de Mokolo.

**Conclusion:**

plusieurs facteurs ont été identifiés et reconnus comme prédicteurs de l´hépatite B conformément à la littérature. Certains facteurs tels que: la profession « Indéterminée » du partenaire et la sérologie de la syphilis sont les résultats spécifiques à cette étude, mais restent à confirmer.

## Abstract (en)

**Introduction:**

perinatal transmission of hepatitis B is responsible for more than one third of cases of viral hepatitis in highly endemic regions. The purpose of this study was to analyse factors associated with hepatitis B infection in pregnant women in the health district of Mokolo/Far North of Cameroon.

**Methods:**

a cross-sectional study was conducted from April 2020 to January 2021. Seven hundred ninety-four consenting pregnant women were selected using quota sampling and convenience sampling in seven health facilities in the health district of Mokolo. Socio-demographic data were collected as well patients´ medical history. Tests to detect hepatitis B serological markers and co-infections (human immunodeficiency virus, syphilis and hepatitis C) were performed. The degree of association with carriage of HbsAg was investigated using the 5% significance level.

**Results:**

after multivariate logistic regression analysis, educational level (OR=1.58; 95% CI=1.08-2.30; p=0.019), partner´s work (OR=4.07; 95% CI=1.33-12.53; p = 0.024), first trimester of pregnancy (OR=2.38; 95% CI=1.12-5.04; p = 0.024) and syphilis serology (OR=3.27; 95% CI=1.29-8.28; p=0.012) were identified as major risk factors. HBsAg seroprevalence was 18.4% (95% CI=15.9-21.3) with HBsAg positivity of 13.7% (95% CI=7.5-19.9) in the health district of Mokolo.

**Conclusion:**

in agreement with the literature, this study showed several factors associated with hepatitis B during pregnancy. Some factors such as « unspecified » partner´s work and positive syphilis serology were specific results of this study, but should be confirmed.

## Introduction

L´hépatite B constitue une réelle préoccupation pour les organisations internationales en général et les pouvoirs publics camerounais en particulier tout comme les autres maladies chroniques transmissibles (VIH, tuberculose) [1]. Selon l´Organisation Mondiale de la Santé (OMS), environ 2 milliards de personnes à travers le monde seraient porteuses de marqueurs sérologiques du virus de l´hépatique B, et 150 millions souffriraient d´hépatite C [2]. En 2015, l´OMS estimait que 257 millions de personnes vivaient avec une hépatite B chronique, dont 65 millions de femmes en âge de procréer avec un nombre de décès estimé à 887 000, principalement du fait de la cirrhose ou du carcinome hépatocellulaire. Elle est particulièrement répandue en Afrique subsaharienne et en Asie de l'Est, affectant 5 à 10% de tous les adultes, tandis qu'en Amérique du Nord, moins de 1% sont infectés. La prévalence estimée est inférieure à 5% en Europe de l'Est, 1,5% en Europe du Nord, 2% en Europe du Sud et 1% en Europe de l'Ouest [3]. Le centre de contrôle et de prévention des maladies a estimé entre 850 000 et 2,2 millions des individus avec une hépatite B chronique aux États-Unis. Environ 25% des enfants et 15% des adultes atteints d´hépatite B chronique décèdent prématurément d´un carcinome hépatocellulaire ou cirrhose [4].

Le VHB est le deuxième carcinogène important après le tabac. La vaccination est systématiquement recommandé aux nouveau-nés car environ 4,5 millions de femmes atteintes de cette affection enfantent chaque année, avec le plus grand nombre en Afrique et dans les régions du pacifique occidental [5]. Une réduction estimée de 16% de ce fardeau serait atteint si une dose du vaccin contre l´hépatite B était systématiquement administrée pour prévenir la transmission périnatale [6]. Le continent africain est tout particulièrement concerné, avec des taux de portage chronique de l´AgHbs élevés, de l´ordre de 15 à 20% dans la population générale et de 22 à 25% chez les femmes enceintes en particulier. Le Cameroun est aussi un pays à forte endémicité, avec une distribution aussi importante en zone rurale (13,3%) qu´en zone urbaine ( 10,7%) [7]. La transmission mère-enfant constitue une voie majeure de transmission du VHB, en particulier dans les zones de forte endémicité [8]. Elle peut avoir lieu selon trois modes distincts à savoir pendant la grossesse (infection intra-utérine), lors de la délivrance et la transmission horizontale post-partum par l'allaitement maternel ou le contact quotidien [9]. La transmission du virus de la mère à l´enfant est due à l´exposition du nouveau-né aux sécrétions maternelles lors de son passage dans la filière génitale ou pendant la période néonatale. La transmission in utero semble rare, représentant 2 à 5% des infections périnatales. Cette transmission verticale est courante chez les femmes porteuses asymptomatiques qui ignorent leur état de porteuse. En cas de multiplication virale élevée chez la mère et en l´absence de sérovaccination, 90% des nouveau-nés infectés sont susceptibles de développer une hépatite chronique B [10] et présentent un risque beaucoup plus élevé de développer une maladie du foie, y compris une cirrhose et un carcinome hépatocellulaire à l'âge adulte [11]. La transmission du VHB de la mère à l´enfant est responsable de plus d´un tiers des hépatites virales chroniques [12]. Une population africaine plus jeune semble être plus affectée, ce qui suggère des taux élevés de transmission maternofœtale [13]. Plusieurs études ont permis de déterminer la prévalence du VHB chez les femmes enceintes. Adegbesan-Omilabu MA *et al*. (2015) ont trouvé une prévalence de 7,3% de l´AgHbs positif chez les femmes enceintes à la clinique prénatale d´un hôpital tertiaire de Lagos, au Nigeria. Les résultats étaient statistiquement significatifs chez celles ayant subi une intervention chirurgicale antérieure, dont un frère ou un conjoint avait été précédemment affecté et chez celles ayant au moins deux partenaires sexuels [14]. Des résultats similaires ont été trouvés chez 7,2% de femmes enceintes à l´hôpital Yirgalem en Éthiopie. Les femmes ayant des antécédents de partenaires sexuels multiples et séropositives au HIV étaient associées de manière significative à la positivité pour l´AgHbs [15]. De même, Völker *et al*. (2017) ont trouvé une prévalence élevée d´infections causées par le virus de l´hépatite B dans une zone rurale du Ghana [16]. Khadidjatou Saké Alassan a observé la pratique des scarifications, l'antécédent personnel d'ictère et les antécédents familiaux d'hépatite virale B chez les femmes gestantes (14,02% de prévalence à l´AgHbs), à Parakou, en République du Bénin [17].

Au Cameroun, plusieurs études ont été effectuées chez les femmes enceintes. À Buea, une prévalence de 9,7% a été constatée avec pour principal déterminant la faible connaissance de la maladie [18]. Une prévalence de 10,2% a été déterminée dans trois autres formations sanitaires et les facteurs de risque étaient la co-infection avec le VIH et les antécédents de transfusion [19]. Chez les femmes qui avaient une prévalence en AgHbe (12,1%), il n´existait pas de transmission périnatale. Dans le district de santé de Tokombéré, la prévalence des femmes enceintes porteuses du VHB était estimé à 18,2% [20]. Njoya et collaborateurs, dans une enquête Connaissances, Aptitudes et Pratiques, ont trouvé une prévalence de 16,11% chez les femmes enceintes. Le faible niveau de connaissances et les mauvaises pratiques étaient les facteurs associés chez ces femmes enceintes. De plus, les personnels médicaux interrogés présentaient une connaissance moyenne associé à de mauvaises pratiques en matière de prévention de la transmission mère-enfant du VHB [21]. Cette transmission est fortement corrélée au statut AgHbe de la mère, avec une transmission, dans 90% des cas, chez les enfants de mères AgHbe positif, contre 10-30% d´enfants de mères AgHbe négatif. La co-infection VIH chez les mères a un impact significatif sur la transmission verticale car, chez celles-ci, la charge virale VHB est élevée [22]. Toutefois, la constatation de l´AgHbs chez des enfants de mères AgHbs négatif et en milieu scolaire suggère qu´il existe une transmission horizontale dans l´enfance au moins égale à la transmission verticale [23].

Pour stopper cette spirale dramatique, il faut diagnostiquer l´infection le plus tôt possible au cours de la grossesse, traiter les femmes enceintes qui présentent une infection évolutive et administrer aux nouveau-nés le vaccin associé dans certains cas aux immunoglobulines anti-Hbs. Il existe des vaccins sûrs et efficaces contre l´hépatite B qui ont été introduits dans le programme élargi de vaccination (PEV) en 2005, avec une couverture de 79% en Pentavalent en 2018 [24]. La prévention de la transmission (services de dépistage et vaccination), la prise en charge et le traitement constituent les stratégies d´élimination de cette infection. Cependant, malgré l´introduction du vaccin dans le PEV et les mesures de prévention, cette prévalence demeure toujours élevée. Il est donc primordial d´identifier d´autres sources de transmission, parmi lesquelles les facteurs pouvant être à l´origine de l´infection. Sachant que les femmes enceintes infectées par le VHB risquent d'infecter leur bébé, il est très important de déterminer l'ampleur du statut VHB et ses facteurs associés parmi cette population de l´Extrême-Nord Cameroun (Mokolo) pour qui, il semble y avoir aucune information à l´heure actuelle.

## Méthodes

**Le cadre de l´étude**: cette étude a été conduite dans cinq formations sanitaires publiques (Centre de santé intégré de Gadala, Centre de santé intégré de Ldamang, Centre de santé intégré de Mokolo I, Centre médical d´arrondissement de Mokolo II et Centre de santé intégré de Zamay-grede) et deux confessionnelles (Centre de santé intégré d´Ouro-tada et Centre de santé intégré de Zamay) du district de santé de Mokolo, département du Mayo-Tsanaga, région de l´Extrême-Nord Cameroun.

**Le Type de l´étude**: une étude quantitative, prospective, transversale et analytique a été menée auprès des femmes enceintes consentantes, fréquentant les FOSA sélectionnées au hasard dans le district de santé de mokolo de novembre à décembre 2020.

**La population d'étude**: un échantillonnage par quota a été fait dans chaque formation sanitaire en tenant compte de la population des femmes enceintes attendues annuellement dans chaque hôpital. Les participantes ont été recrutées de manière consécutive et exhaustive au fur et à mesure qu´elles arrivaient à la consultation prénatale au sein des formations sanitaires sélectionnées durant la période d´étude.

**Échantillon de l´étude**: la taille de l´échantillon a été calculée par la formule de Cochran [25] utilisant une prévalence de 18,2% chez les femmes enceintes [20]:


N=(Z1−α/2)2*p*(1−p)e2

Avec Z_1-α/2_= 1,96 qui est le constant de la distribution normale pour une valeur α = 5%; p = prévalence de l´évènement étudié dans la population et e = niveau de précision qui a été fixé à 3%.

**Outils et procédure de collecte de données**: l´outil de collecte des données utilisé dans cette étude était un questionnaire pré-testé conçu pour les femmes enceintes.

**Prélèvement et analyse au laboratoire des échantillons sanguins**: des échantillons de cinq ml de sang veineux ont été prélevés à l´aide d´une séringue de 10 ml et conservés dans un tube d'acide éthylène diamine tétra acétique (EDTA). Chaque échantillon était étiqueté en fonction des codes des registres de consultations prénatales des formations sanitaires sélectionnées et stocké dans une glacière contenant des accumulateurs et transporté au laboratoire de l´hôpital régional annexe de Mokolo en fin de journée. Chaque tube contenant du sang veineux total était centrifugé afin de séparer le plasma du culot globulaire par la technique de centrifugation à la température ambiante de 3000 tr/min [26]. Ce plasma était ensuite recueilli à l´aide d´une micropipette et d´un embout bleu dans le cryotube. Chaque cryotube étiqueté avec le même code de départ était conservé à une température comprise entre 2°C et 8°C lorsque la recherche n´était pas immédiate pour les marqueurs sériques de l´hépatite B (HBV 5-in-1 ONE STEP: l´AgHbs, l´AgHbe, AcHbc), les co-infections (VIH (Alere Determine HIV-1/2), la syphilis (test SD Bioline Syphilis 3.0) et VHC (HCV Rapid Test Strip (Sérum/Plasma)). Pour maintenir la qualité des résultats de laboratoire, les procédures opérationnelles standard (SOP) du fabricant du kit de test ont été strictement suivies.

**Méthode d´analyse des données**: les données ont été saisies dans le logiciel CSPRO version 7.3 puis exportées dans le logiciel SPSS version 26 pour l´analyse statistique. Les tableaux ont été édités à l´aide d´Office Word 2013. Des statistiques sommaires telles que les fréquences, les pourcentages, les médianes et quartiles ont été calculés. Une analyse de régression logistique a été utilisée pour déterminer l'association entre les variables explicatives et la variable de résultat avec un rapport de cotes à 95% IC. Toutes les variables explicatives avec une valeur de p < 0,05 dans l'analyse univariée ont été incluses dans le modèle de régression logistique multivariée. La valeur p < 0,05 en analyse multivariée a été considérée comme une signification statistique. Le rapport de cotes avec un IC à 95% a été utilisé pour mesurer la force de l'association. Pour le rapport de cote, une variable indépendante (VI) était considérée comme: 1) facteur de risque: si la VI du modèle avait un rapport de cote supérieur à 1; 2) facteur de protectionsi la VI du modèle de régression avait un rapport de cote compris entre 0 et 1.

**Considérations éthiques**: sur le plan du respect des considérations éthiques dans cette étude, un certificat éthique portant le N° 2020/02060/CEIRSH/ESS/MSP a été délivré par le comité éthique de l´École des Sciences de la Santé de l´Université Catholique d´Afrique Centrale. Après cette approbation du comité, des autorisations d´enquête ont été successivement obtenues auprès de la délégation régionale de la santé de l´Extrême-Nord, du district de santé de Mokolo et au niveau des FOSA. Pour chaque participante, l´objet et la démarche de la recherche ont été clairement expliqués. Leur consentement était recueilli à l´aide de la notice d´information et de la fiche du consentement éclairé, destinée à cet effet avant leur introduction dans la population.

## Résultats

**Facteurs sociodémographiques associés à la prévalence de l´hépatite B chez les femmes enceintes**: au total, 794 femmes enceintes ont participé à cette étude avec un taux de réponse de 100%. L´âge médian de la population d´étude était de 25 ans avec un intervalle interquartile de 20-30 ans; les valeurs extrêmes étaient 15 et 48 ans. Le nombre médian de foyer en cas de polygamie était de 2 (2-3) avec les extrêmes de 2 et 6 foyers. Dans cette étude, la prévalence de l´antigène de l´hépatite B (AgHbs) était 18,4% (146/794 ) et celle de l´AgHbe était de 13,7% (20/146). En analyse univariée, la tranche d´âge ≥30 ans (OR = 1,50; p = 0,038) et le niveau d´étude « Aucun » (OR = 1,58; p = 0,012) étaient significativement associés à la présence de l´AgHbs. Après analyse multivariée, les femmes enceintes qui avaient le niveau d´étude “aucun” étaient 1,6 fois plus susceptible d'être infectée par le VHB que les autres niveaux d´étude (Tableau 1).

**Tableau 1:** variation de la prévalence de l´hépatite B selon les caractéristiques socio-démographiques des femmes enceintes

Variables	Présence de l´AgHbs			P Brut	OR (IC à 95 %) brut	P ajusté	OR (IC à 95 %) ajusté
	Oui n (%)	Non n (%)	Total n (%)				
**Age (en années)**							
< 20	18 (13,2)	118 (86,8)	136 (100)	0,088	0,63 (0,37-1,08)		
[20-29[	79 (17,7)	367(82,3)	446 (100)	0,578	0,90 (0,63-1,30)		
≥ 30	49 (23,1)	163 (76,9)	212 (100)	**0,038**	**1,50 (1,02-2,21)**	0,075	1,45 (0,96-2,17)
**Statut matrimonial**							
Mariée monogame	93 (17,2)	447 (82,8)	540 (100)	0,216	0,79 (0,54-1,15)		
Mariée polygame	49 (21,6)	178(78,4)	227 (100)	0,141	1,33 (0,91-2,00)		
Célibataire	4(14,8)	23(85,2)	27 (100)	0,802	0.77 (0,26-2.25)		
**Niveau d´étude**							
Aucun	80 (22,2)	281(77,8)	361 (100)	**0,012**	**1,58 (1,10-2,27)**	**0,019**	1,58 (1,08-2,30)*
Primaire	34 (14,3)	204 (85,7)	238 (100)	0,051	0.661 (0,44-1,00)		
Secondaire	32 (16,4)	163 (83,6)	195 (100)	0,412	0,84 (0,54-1,28)		
**Profession**							
Agricultrice	62 (19,0)	264 (81,0)	326 (100)	0,702	1,07 (0,75-1,54)		
Femme au foyer	68 (18,7)	295 (81,3)	363 (100)	0,818	1,04 (0,73-1,50)		
Commerçante	6 (15,8)	32 (84,2)	38 (100)	0,672	0,83 (0,34-2,01)		
Autre	10 (14,9)	57 (85,1)	67 (100)	0,445	0,76 (0,38-1,53)		
**Réligion**							
Musulmane	17 (12,9)	115 (87,1)	132 (100)	0,074	0,61 (0,35-1,05)		
Chrétienne	112(19,6)	459 (80,4)	571 (100)	0,153	1,36 (0,89-2,06)		
Animiste	17 (18,7)	74 (81,3)	91 (100)	0,939	1,02 (0.58-1,79)		
**Ethnie**							
Mafa	98 (19,8)	397 (80,2)	495 (100)	0,187	1.29 (0,88-1,88)		
Non mafa	48 (16,1)	251 (83,9)	299 (100)				

**Facteurs environnementaux associés à la prévalence de l´hépatite B chez les femmes enceintes**: le nombre médian de personnes vivant dans le même ménage était de 6 (4-9), avec les extrêmes de 2 et 40 personnes. L´âge médian du partenaire était de 34 ans avec un intervalle interquartile de 30-40 ans; et les valeurs extrêmes de 19 et 80 ans. En analyse univariée, la profession « Indeterminé » (OR = 3,43; p = 0,029) était associée de façon statistiquement significative à la présence de l´AgHbs. Après analyse multivariée, les femmes enceintes qui avaient un partenaire ayant une profession “ Indéterminée ” étaient 4 fois plus susceptible d'être infectée par le VHB (Tableau 2).

**Tableau 2:** variation de la prévalence de l´hépatite B selon les caractéristiques socio-démographiques des partenaires des femmes enceintes

Variables	Présence de l´AgHbs			P brut	OR (IC à 95 %) brut	P ajusté	OR (IC à 95 %) ajusté
	Oui n (%)	Non n (%)	Total n (%)				
**Age (en années)**							
< 20	1 (11,1)	8 (88,9)	9 (100)	0,571	0,55 (0,68-4,44)		
[20-29[	32 (18,0)	146 (82,0)	178 (100)	0,873	0,97 (0,63-1,49)		
[30-39[	72 (17,9)	331 (82,1)	403 (100)	0,700	0,93 (0,65-1,33)		
[40-49[	34 (21,7)	123 (78,3)	157 (100)	0,238	1,30 (0,84-1,99)		
≥ 50	7 (14,9)	40 (85,1)	47 (100)	0,524	0,77 (0,34-1,74)		
**Niveau d´étude**							
Aucun	45 (19,2)	189 (80,8)	234 (100)	0,692	1,08 (0,73-1,60)		
Primaire	38 (17,7)	177 (82,3)	215 (100)	0,752	0,94 (0,62-1,41)		
Secondaire	51 (17,1)	248 (82,9)	299 (100)	0,452	1,16 (0,79-1,68)		
Je ne sais pas	12 (26,1)	34 (73,9)	46 (100)	0,165	1,62 (0,82-3,21)		
**Profession**							
Agriculteur	89 (20,5)	346 (79,5)	435 (100)	0,097	1,36 (0,95-1,97)		
Commerçant	18 (16,8)	89 (83,2)	107 (100)	0,653	0,88 (0,51-1,52)		
Fonctionnaire	10 (13,2)	66 (86,8)	76 (100)	0,216	0,65 (0,33-1,29)		
Indéterminée	6 (42,9)	8 (57,1)	14 (100)	**0,029**	**3,43 (1,17-10,3)**	**0,014**	4,07 (1,33-12,53)*
Autre	23(14,2)	139 (85,8)	162 (100)	0,123	0,69 (0,42-1,11)		

**Facteurs comportementaux associés à la prévalence de l´hépatite B chez les femmes enceintes**: parmi les caractéristiques comportementales, aucune variable n´était associée à la prévalence de l´hépatite B (Tableau 3).

**Tableau 3:** variation de la prévalence de l´hépatite B en fonction des caractéristiques comportementales des femmes enceintes

Variables	Présence de l´gHbs			P Brut	OR (IC à 95 %) Brut
	Oui n (%)	Non n (%)	Total n (%)		
**Partage de rasoirs (oui)**	66 (20,0)	264 (80,0)	330 (100)	0,323	1,20 (0,84-1,72)
**Partage de brosses à dent (oui)**	6 (26,1)	17 (73,9)	23 (100)	0,333	1,59 (0,62-4,11)
**Aviez-vous entendu parler de l´hépatite B ? (oui)**	31 (14,8)	178 (85,2)	229 (100)	0,122	0,71 (0,46-1,10)
**Nombre de partenaires sexuels au cours des 3 derniers mois (un seul)**	145 (18,3)	647 (81,7)	792 (100)	0,334	0,22 (0,14-3,60)
**Consommation de la drogue (oui)**	5 (17,2)	24 (82,8)	29 (100)	0,871	0,92 (0,35-2,46)
**Alcoolisme (oui)**	62 (21,6)	225 (78,4)	287 (100)	0,079	1,39 (0,96-2,00)
**Tatouage (oui)**	0 (0,0)	5 (100,0)	5 (100)	0,591	N.A
**Scarification (oui)**	21 (25,0)	63 (75,0)	84 (100)	0,098	1,56 (0,92-2,65)
**Vaccination contre le virus de l´hépatite B(oui)**	1(6,7)	14 (93,3)	15 (100)	0,237	0,31 (0,04-2,39)

**Facteurs liés au système de sante associés à la prévalence de l´hépatite B chez les femmes enceintes**

**Caractéristiques cliniques associés à la prévalence de l´hépatite B chez les femmes enceintes**: parmi les caractéristiques cliniques, aucune variable n'était associée à la prévalence de l´hépatite B (Tableau 4).

**Tableau 4:** variation de la prévalence de l´hépatite B selon les caractéristiques cliniques des femmes enceintes

Variables	Présence de l´AgHbs			P brut	OR (IC à 95 %) Brut
	Oui n (%)	Non n (%)	Total n (%)		
**Antécédents familiaux de VHB (oui)**	3 (23,1)	10 (76,9)	13 (100)	0,711	1,39 (0,37- 5,18)
**Antécédents d´opérations chirurgicales (oui)**	5 (29,4)	12 (70,6)	17 (100)	0,218	1,88 (0,65-5,42)
**Soins dentaire antérieures (oui)**	11 (22,0)	39 (78,0)	50 (100)	0,496	1,27 (0,64-2,55)
**Notion de transfusion sanguine dans le passé (oui)**	7 (30,4)	16 (69,6)	23 (100)	0,166	1,99 (0,80-4,93)
**Notion d´excision (oui)**	7 (22,6)	24 (77,4)	31(100)	0,539	1,31 (0,55-3,10)
**Statut VIH/SIDA (positif)**	2 (40,0)	3 (60,0)	5 (100)	0,196	3,38 (0,56-20,51)
**Antécédent d´infection sexuellement transmissible (oui)**	11 (21,6)	40 (78,4)	51 (100)	0,356	1,40 (0,68-2,86)
**Séance d´IEC sur la PTME de l´hépatite virale B (oui)**	38 (18,4)	168 (81,6)	206 (100)	0,980	1,00 (0,67-1,52)
**Offre globale des examens obligatoires de la CPN (oui)**	126 (18,3)	564 (81,7)	690 (100)	0,812	0,94 (0,56-1,59)

**Caractéristiques obstétricales associées à la prévalence de l´hépatite B chez les femmes enceintes**: l´âge gestationnel médian de la population d´étude était de 28 semaines avec un intervalle interquartile de 21-32, et les valeurs extrêmes de 4 et 40 semaines. Le nombre médian de CPN était de 3 (2-4), avec les extrêmes de 1 et 9 consultation (s). En analyse univariée, l´âge gestationnel médian (OR = 1,51; p = 0,033), le premier trimestre de la grossesse (OR = 2,78; p = 0,03) et le troisième trimestre (OR = 0,65; p = 0,025) étaient associés de façon statistiquement significative à la présence de l´AgHbs. Après analyse multivariée, les femmes enceintes qui étaient au premier trimestre de grossesse étaient 2,8 fois plus susceptibles d'être infectées par le VHB (Tableau 5).

**Tableau 5:** variation de la prévalence de l´hépatite B selon les caractéristiques obstétricales des femmes enceintes

Variables	Présence de l´AgHbs			P Brut	OR (IC à 95 %) Brut	P ajusté	OR (IC à 95 %) ajusté
	Oui n (%)	Non n (%)	Total n (%)				
**Age Gestationnel Médiane**							
< 28 semaines	100 (20,7)	382 (79,3)	482 (100)	**0,033**	**1,51 (1,03-2,22)**	1,000	1.00 (0,16-6,05)
≥ 28 semaines	46 (14,7)	266 (85,3)	312 (100)				
**Stade de la grossesse**							
1^er^ trimestre	13 (37,1)	22 (62,9)	35 (100)	**0,003**	**2,78 (1,37-5,66)**	**0,024**	2,38 (1,12-5,04)*
2^e^ trimestre	86 (19,6)	352 (80,4)	438 (100)	0,314	1,21 (0,84-1,74)		
3^e^ trimestre	47 (14,6)	274 (85,4)	321 (100)	**0,025**	**0,65 (0,44-0,95)**	0,641	0,66 (0,11-3,91)
**Nombre de CPN Médiane**							
< 3	106 (20,3)	416 (79,7)	522 (100)	0,053	1,48 (1,00-2,22)		
≥ 3	40 (14,7)	232 (85,3)	272 (100)				
**Parité**							
Nullipare	27 (15,7)	145 (84,3)	172 (100)	0,303	0,79 (0,50-1,24)		
Multipare	119 (19,1)	503 (80,9)	622 (100)				
**Antécédents d´avortement (oui)**	18 (15,8)	96 (84,2)	114 (100)	0,439	0,81 (0,47-1,39)		
**ATCD de césarienne (oui)**	3 (33,3)	6 (66,7)	9 (100)	0,244	2,25 (0,56-9,08)		
**ATCD D´accouchement instrumental (oui)**	3 (15)	17 (85)	20 (100)	1,000	0,78 (0,23-2,69)		

**Les co-infections (VHC, VIH et Syphilis) associées à la prévalence de l´hépatite B en fonction chez les femmes enceintes**: la prévalence des co-infections variait en fonction de l´infection. Au total neuf femmes (6,2%) étaient co-infectées au VHC et au VHB, une seule femme (0,7%) au VIH et au VHB, et neuf femmes (6,2%) à la syphilis et au VHB. En analyse univariée, la sérologie positive de la syphilis (OR = 3,8; p = 0,005) était associée de façon statistiquement significative à la présence de l´AgHbs. Après analyse multivariée, les femmes enceintes qui avaient une sérologie positive à la syphilis étaient 3,3 fois plus susceptibles d'être infectées par le VHB (Tableau 6).

**Tableau 6:** variation de la prévalence de l´hépatite B en fonction des co-infections

Variables	Présence de l´AgHbs			PBrut	OR (IC à 95 %) Brut	Pajusté	OR (IC à 95 à %) ajusté
	Oui n (%)	Non n (%)	Total n ( %)				
**Anti-VHC**							
Positif	9 (15)	51 (85)	60 (100)	0,481	0,77 (0,37-1,60)		
Négatif	137 (18,7)	597 (81,3)	734 (100)				
**Sérologie VIH**							
Positif	1 (10)	9 (90)	10 (100)	0,699	0,49 (0,62-3,90)		
Négatif	144 (18,4)	638 (81,6)	782 (100)	1,000	1,13 (0,25-5,21)		
Inconnu	1 (50)	1 (50)	2 (100)	0,334	4,46 (0,28-71,76)		
**Sérologie Syphilis**							
Positif	9 (45)	11 (55)	20 (100)	**0,005**	**3,80 (1,55-9,34)**	**0,012**	3,27 (1,29-8,28)*
Négatif	137 (17,7)	637 (82,3)	774 (100)				

## Discussion

La prévalence de l´AgHbs chez les femmes enceintes dans le district de santé de Mokolo était de 18,4%. Ce résultat confirme les projections du Ministère de la santé du Cameroun (2020, p.23) qui classe l´Extrême-Nord comme une région à forte prévalence. Cette prévalence est similaire à celle rapportée par Loriette *et al*. (2015) (18,2%) [20] dans la zone de Tokombéré. En revanche, elle est supérieure à celles trouvées dans les districts de Buea (9,7%) par Frambo *et al*. (2014) [18] et Guidiguis (10,2%) par Noubiap *et al*. (2015) [19]. Ces différences pourraient être liées au fait que les districts de Tokombéré et Mokolo sont des villages situés à proximité de zones politiquement instables du Nigeria et que la nature du district (rural et montagneux) rend difficile l'accès aux établissements de santé.

La prévalence de l´AgHbe chez les femmes enceintes ayant un AgHbs positif était de 13,7% (7,5-19,9%). Ce résultat est proche de celui trouvé par Noubiap *et al*. ( 2015) [19], qui était de 12,1%. En revanche, cette prévalence est inférieure à celle trouvée par Ducancelle *et al*. (2013) [27] qui était de 22,7%. L´explication à cette différence n´a pas été trouvée mais la positivité de l´AgHbe augmentait le risque de transmission périnatale. La tranche d´âge ≥ 30 ans multipliait le risque de portage de l´AgHbs de 1,5 fois chez les femmes enceintes. Par contre les précédentes études ont rapporté que l´âge n´était pas associé à la présence de l´AgHbs [19,28,29]. Cette différence pourrait être liée à la taille de l´échantillon. Le niveau d´étude « Aucun » chez les femmes enceintes multipliaient le risque de portage de l´AgHbs de 1,5 fois. Abongwa *et al*. (2016), Metaferia *et al*. (2016) ont aussi trouvé un lien entre le niveau d´instruction et le portage de l´AgHbs [29,30]. Par contre, Ousmane *et al*. (2018), Koh *et al*. (2019) n´ont pas trouvé un lien statistique entre le niveau d´instruction et le portage de l´AgHbs [31,32]. Ces différences pourraient être liées à la taille de l´échantillon et au fait de l´ignorance des consultations prénatales chez les femmes enceintes en zone rurale par rapport à la zone urbaine. Le nombre de partenaires sexuels n´était pas statistiquement associé au portage de l´AgHbs. Par contre, plusieurs études ont relevé que le fait d´avoir plusieurs partenaires sexuels était statistiquement associé au portage chronique de l´AgHbs (Adegbesan-Omilabu *et al*. 2015; Koh *et al*. 2019; Amsalu *et al*. (2018) [14,15,32]. Cette différence pourrait être liée à la taille de l´échantillon et à la population de l´étude.

La scarification n´était pas associée de façon statistiquement significative au portage de l´AgHbs. Ce résultat était similaire à celui de Noubiap *et al*. (2015) [19]. Par contre, plusieurs autres auteurs comme Alassan *et al*. (2019), Koh *et al*. (2019), ont trouvé que la pratique de la scarification était un facteur de risque significatif [17,32]. Cette différence pourrait être liée à la puissance de l´étude. Parmi les caractéristiques cliniques, aucune variable n'était associée à la prévalence de l´hépatite B. Koh *et al*. (2019) ont trouvé dans une étude cas-témoins que l´antécédent de transfusion n´était pas associé au portage de l´AgHbs, similairement à la nôtre [32]. Par contre, plusieurs études ont identifié les caractéristiques cliniques associées à l´AgHbs. Noubiap *et al*. (2015), Ousmane *et al*. (2018), ont observé une association significative entre l´antécédent de transfusion sanguine et le portage de l´AgHbs [19, 31]. De même, l´antécédent familial de l´hépatite B a été associé à la présence de l´AgHbs [17,32]. Les soins dentaires et les antécédents de chirurgie étaient associés au portage de l´AgHbs [32]. Ces différences pourraient être liées à la puissance de l´étude, au type d´étude (l´étude cas-témoins à une précision supérieure à l´étude transversale), au lieu de l´étude (urbanisation), à la population d´étude, aux caractéristiques socioculturelles (réticence dans le cadre de la transfusion) et à la transfusion non sécurisée, ainsi qu´à la fenêtre sérologique lors des tests de transfusion sanguine. L´âge gestationnel médian multipliait le risque de portage de l´AgHbs de 1,5 fois. De même, le premier trimestre de la grossesse multipliait le risque de portage de l´AgHbs de 2,7 fois. Par contre, le troisième trimestre de la grossesse était un facteur de protection et diminuait le risque de 35% de portage de l´AgHbs chez les femmes enceintes.

Giles *et al*. (2015) avaient trouvé que le stade de la grossesse était statistiquement lié au portage de l'AgHbs, similairement à cette étude [33]. Par contre, Amsalu *et al*. (2018) n´ont trouvé aucun lien entre le stade de la grossesse et le portage de l'AgHbs [15]. Plusieurs auteurs ont trouvé que la parité chez les femmes enceintes n'était pas associée au portage de l'AgHbs ainsi que dans cette étude [15,29]. Par contre, Giles *et al*. (2015) ont montré que la parité et le portage de l'AgHbs avaient un lien statistique [33]. Cette différence pourrait être liée aux caractéristiques de la population d´étude (femmes enceintes atteintes de l´hépatite chronique) et au type d´étude (cohorte). L´antécédent d´avortement dans cette étude n´a pas de lien statistique au portage de l'AgHbs similaire à celui de Noubiap *et al*. (2015) et Amsalu *et al*. (2018) [15,19]. Cette similitude pourrait être liée aux caractéristiques socioculturelles (l´avortement est considéré comme un crime) des femmes enceintes dans ces études. La co-infection au VIH n´était pas un facteur associé au portage de l´AgHbs dans cette étude similaire à celle de Koh *et al*. (2019) [32]. Par contre, Noubiap *et al*. (2015) et Amsalu *et al*. (2018) ont trouvé une relation statistiquement significative entre la co-infection au VIH et le portage de l´AgHbs [15,19]. Ces différences pourraient être liées à la puissance de l´étude, au type d´étude et à l´absence de comportements à risque dans cette étude. Une rigueur méthodologique dans cette étude a été effective, mais il est important de noter quelques limites identifiées. Tout d´abord, le caractère quantitatif transversal analytique de cette étude n´a pas permis de prouver les associations de causalité comme le ferait une étude cas-témoins. Une étude cas-témoins a un degré de preuve de causalité plus approfondie que les études transversales analytiques. Aussi, certaines caractéristiques comme la consommation de l´alcool, l´utilisation de la drogue et le statut du conjoint par rapport à l´AgHbs n´ont pas été pris en compte dans le processus de collecte des données. L'utilisation des tests de diagnostic rapide commercial, qui sont moins sensibles que le test ELISA ou la PCR, conduisent à une sous-estimation possible de la prévalence des marques évaluées. Au niveau des participants, un biais de rappel des informations sur la recherche des facteurs associés chez les femmes enceintes a été constaté. La recherche de l´anticorps Hbe et le dosage de la charge virale du VHB n´ont pas pu être réalisés alors qu´ils sont des déterminants importants dans le diagnostic de l´hépatite B.

## Conclusion

En somme, cette étude qui a eu pour objectif d´analyser les facteurs associés à l´hépatite B chez les femmes enceintes dans les formations sanitaires du district de santé de Mokolo/région de l´Extrême-Nord Cameroun a permis d´avoir la prévalence de l´AgHbs dans le district de santé de Mokolo estimée à 18,4%, avec une positivité à l´AgHbe de 13,7% faisant d´elle une zone d´endémicité élevée selon la classification de l´OMS. La prévalence des co-infections était de 6,2% entre VHC et VHB, de 0,7% entre VIH et VHB et de 6,2% entre la syphilis et VHB. En analyse univariée, des facteurs tels que: la tranche d´âge ≥ 30 ans, le niveau d´étude « Aucun », la profession « Indéterminée », l´âge gestationnel médian, le premier trimestre de la grossesse, le troisième trimestre de grossesse et la sérologie positive de la syphilis étaient associés de façon statistiquement significative à la présence de l´AgHbs. Après l´analyse multivariée, le niveau d´étude de la femme enceinte (Aucun), la profession « Indéterminée » du partenaire, le premier trimestre de la grossesse et la sérologie syphilis positive étaient des facteurs de risque persistants. L´implication de la profession « Indéterminée » du partenaire et la sérologie de la syphilis « positive », qui sont des résultats spécifiques, restent à confirmer par des études ultérieures.

## Remerciements

Nous souhaitons remercier les personnels du Laboratoire de l´Hôpital Régional Annexe de Mokolo, les chefs des centres de santé et le chef de district de santé de Mokolo, pour leur coopération et leur soutien au cours de cette étude.
